# CD44 In Sarcomas: A Comprehensive Review and Future Perspectives

**DOI:** 10.3389/fonc.2022.909450

**Published:** 2022-06-17

**Authors:** Enrique Fernández-Tabanera, Raquel M. Melero-Fernández de Mera, Javier Alonso

**Affiliations:** ^1^ Unidad de Tumores Sólidos Infantiles, Instituto de Investigación de Enfermedades Raras (IIER), Instituto de Salud Carlos III (ISCIII), Madrid, Spain; ^2^ Centro de Investigación Biomédica en Red de Enfermedades Raras, Instituto de Salud Carlos III (U758; CB06/07/1009; CIBERER-ISCIII), Madrid, Spain; ^3^ Universidad Nacional de Educación a Distancia (UNED), Madrid, Spain

**Keywords:** CD44, sarcoma, cancer therapy, biomarker, CD44-ICD, signalling transduction, epithelial mesenchymal transition (EMT), extracellular matrix (ECM)

## Abstract

It is widely accepted that the tumor microenvironment, particularly the extracellular matrix, plays an essential role in the development of tumors through the interaction with specific protein-membrane receptors. One of the most relevant proteins in this context is the transmembrane protein CD44. The role of CD44 in tumor progression, invasion, and metastasis has been well established in many cancers, although a comprehensive review concerning its role in sarcomas has not been published. CD44 is overexpressed in most sarcomas and several *in vitro* and *in vivo* experiments have shown a direct effect on tumor progression, dissemination, and drug resistance. Moreover, CD44 has been revealed as a useful marker for prognostic and diagnostic (CD44v6 isoform) in osteosarcoma. Besides, some innovative treatments such as HA-functionalized liposomes therapy have become an excellent CD44-mediated intracellular delivery system for osteosarcoma. Unfortunately, the reduced number of studies deciphering the prognostic/diagnostic value of CD44 in other sarcoma subgroups, neither than osteosarcoma, in addition to the low number of patients involved in those studies, have produced inconclusive results. In this review, we have gone through the information available on the role of CD44 in the development, maintenance, and progression of sarcomas, analyzing their implications at the prognostic, therapeutic, and mechanistic levels. Moreover, we illustrate how research involving the specific role of CD44 in the different sarcoma subgroups could suppose a chance to advance towards a more innovative perspective for novel therapies and future clinical trials.

## 1 Introduction

Sarcomas are a heterogeneous group of tumors originating from mesenchymal cells. These rare malignancies account for approximately 10% of childhood solid tumors arising in soft and bone tissues (1), and 2% of cancers diagnosed in adult individuals (representing an overall annual incidence of approximately six adult cases per 100,000 people in Europe) (2, 3). Sarcomas are mostly detected in the extremities (approximately 60%) and the trunk (around 18%) (4), but they can arise from any part of the body (retroperitoneum-12%, head and neck-9%, and mediastinum-1%). Metastases, when detected, are located predominantly in the lung, although are rarely present at the time of diagnosis (around 10% of the cases) (5–7).

According to the World Health Organization (WHO), there are more than 100 histological subtypes of sarcomas with different clinical characteristics (8). This classification considers the histology but also key genetic alterations, which are found within specific sarcomas. During the last decades, our understanding of the molecular mechanisms that underlie most of the sarcomas has been largely determined, contributing to improving diagnosis and treatment. The genetic alterations more frequently detected in sarcomas are: tyrosine-kinase activating mutations (9), gene fusions of growth factors or kinases (e.g., involving ALK, ROS1 or NTRK family) (10–12), gene fusions involving transcriptions factors (e.g., EWSR1-FLI1, PAX3-FOXO1) (13, 14), inactivation of tumor suppressor genes (e.g., NF1, PTEN or TP53) (15–17), gene amplification (e.g. MDM2 and MDM2/CDK4 co-amplification) (18) and epigenetic dysregulation (19, 20).

In addition to genetic alterations, other factors are also involved in the initiation, maintenance, and progression of cancer cells (21). Thereby, it is now widely accepted that the tumor microenvironment plays an essential role in tumor maintenance and progression. Particularly, the extracellular matrix (ECM) regulates many aspects related to the processes of cancer cell invasion, cancer cell dissemination, and the establishment of distant foci of metastasis. The components of the ECM interact with specific protein membrane receptors activating signaling pathways involved in migration, epithelial-mesenchymal transition (EMT), mesenchymal-epithelial transition (MET), and stemness (22). Collagens, proteoglycans, laminins, fibronectins, glycosaminoglycans, as the hyaluronic acid, or matricellular proteins, as periostin, are extracellular components present in the ECM. All of them are able to interact with specific receptors modulating the malignant phenotype, the metastatic processes, or the resistance to drugs (23, 24). One of the more relevant actors in this scenario is the transmembrane protein CD44, which interacts with many ECM components, triggering multiple signaling cascades within tumor cells. The role of CD44 in tumor progression and particularly in the processes of invasion and metastasis has been well established in many cancers mainly of epithelial origin, which have been the subject of excellent reviews (25–27). However, as far as we know, no comprehensive review compiling the data concerning the role of CD44 in sarcomas has been published. In this review, we have gone through the information available on the role of CD44 in the development, maintenance, and progression of sarcomas and analyzed their implications at the prognostic, therapeutic, and mechanistic levels.

## 2 CD44: Structure, Ligands, and Signaling

### 2.1 CD44 Structure

**Figure 1:**
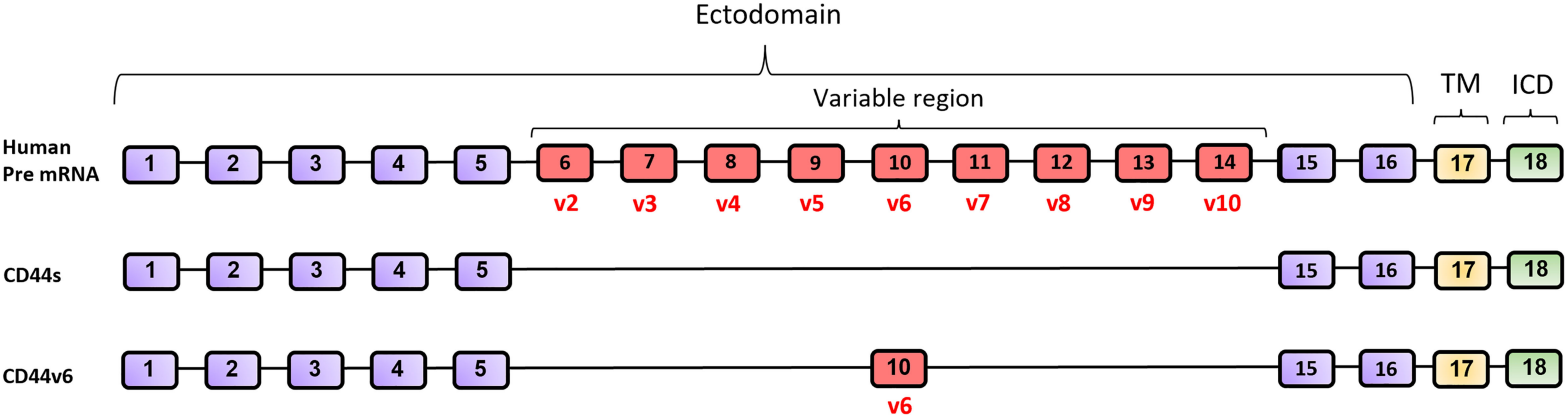
Diagram of full-length CD44 pre-mRNA, CD44s and CD44v6 mRNA in human. CD44 is encoded by 18 exons (ENSEMBL accession number #ENSG00000026508. https://www.ensembl.org/). CD44 isoform 1 (CD44v1) is not present in humans. Exons marked in purple color are always expressed in the ectodomain of all CD44 isoforms. Up to nine exon variants can be inserted by alternative splicing to compose the variable region (Exons 6-14 in red). Exon 17 (yellow) codes for the transmembrane domain (TM) and exon 18 (green) codes for the intracellular domain (ICD).

**Figure 2:**
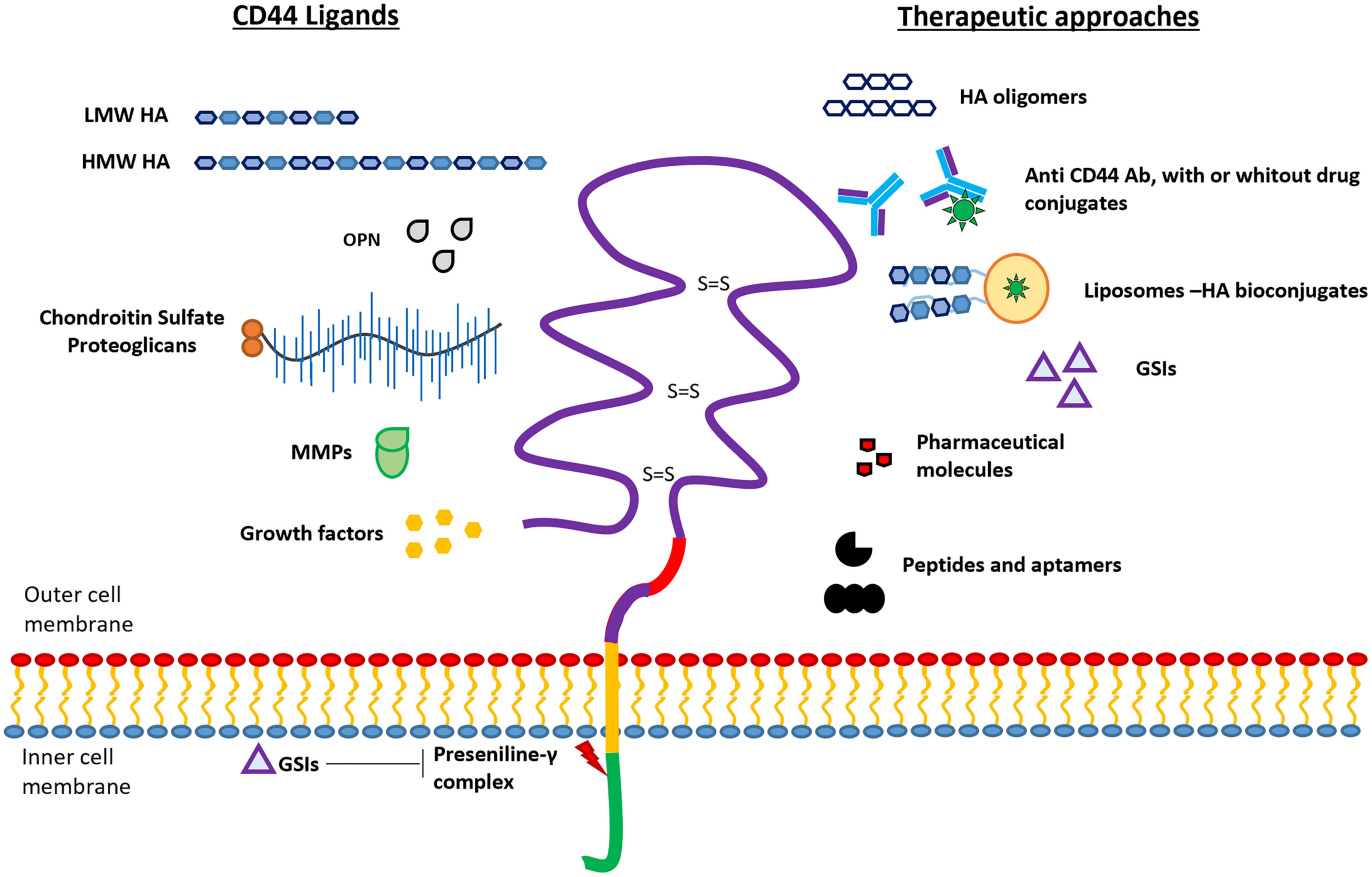
Schematic representation of CD44 protein structure. The four domains of CD44 glycoprotein are represented with different colors: ectodomain (purple), variable domain (red), transmembrane domain (yellow), and intracellular domain (green). Ligands interacting with the ectodomain (left), and the different therapeutic approaches (right) are schematically represented. Presenilin‐γ complex promotes the ICD cleavage which can be blocked by using γ-secretase inhibitors (GSIs).

The gene encoding human CD44 protein is located on the short arm of chromosome 11 and it is composed of 18 exons (HGNC:1681). Several exons (exons 6-14) undergo alternative splicing generating different isoforms (CD44v2-v10) (**Figure 1**) (25, 28). The isoform CD44v1 is not present in humans due to the absence of one exon in comparison to the CD44 gene in mice, which is composed of 19 exons instead 18 (28). The CD44 standard isoform, called CD44s (29), is composed of exons 1-5 (N-terminal and ligand binding domain) and exons 15-18 (C-terminal domain), thus excluding all the alternatively spliced exons. There are three differentiated regions in this complex cell-surface glycoprotein (**Figure 2**): i) the ectodomain, constituted by exons 1-16. It includes the alternatively spliced regions and thus their length is variable. This domain interacts with numerous extracellular ligands; ii) the transmembrane domain (TM, exon 17) and iii) the intracellular domain (ICD, exon 18) which interacts with kinases and other signaling molecules (27, 30) (UniProt accession number: P16070).

### 2.2 CD44 Ligands

The extracellular domain of CD44 has been shown to interact with several ligands as for example hyaluronic acid (HA), osteopontin (OPN), serglycin/sulfated proteoglycans, fibronectin, collagen, and matrix metalloproteases (MMPs) (**Figure 2**). The binding of these ligands to CD44 triggers diverse cellular signaling cascades, some of which have been studied in sarcomas (see below) (25, 27, 31–37). The bioavailability of these biomolecules at the tumor or metastatic sites can modulate cancer progression through the activation of specific pathways. In this section, we analyzed briefly the effects triggered by CD44-ligand interactions.

#### 2.2.1 Hyaluronic Acid (HA)

HA is a primary ECM constituent (commonly found in connective tissue and bone marrow) and the major ligand for CD44 (36). HA is a linear polymer of disaccharide units of β-1,3-N-acetyl glucosamine and β-1,4-glucuronic acid that is synthesized by the hyaluronan synthase proteins (HAS1-3) (38). The CD44/HA binding region resides in the N-terminal domain, located in residues 21-178, and is stabilized by three cysteine disulfide cross-links (39) (**Figure 2**). Interestingly, the molecular weight of HA, which can vary between dozens (≈5 KDa) and thousands of units (2,000 KDa), affects the binding of HA to CD44 and consequently to the effects that it causes in tumor cells (35). High molecular weight HA (HMW-HA, >500kDa) causes cell cycle arrest and reduces proliferation, neo-angiogenesis, and inflammation (35, 40), while low molecular weight HA (LMW-HA, 1-500 kDa) induces proliferation, ECM degradation processes (mediated by metalloproteases) and cell migration and invasion (35, 41). Ras, MAPK, PI3K, Nanog‐Stat3, Oct4‐Sox2‐Nanog, c-Met or c‐Src kinase signaling are some of the pathways directly modulated through CD44-HA binding (42–45). Merlin-ERM (ezrin-radixin-moesin) family of proteins, acts as a tumor suppressor by inhibiting the CD44-HA signaling (46).

#### 2.2.2 Osteopontin (OPN)

OPN is an acidic phosphoprotein mainly secreted in plasma by macrophages and activated T cells and into the bone matrix by osteoblasts, among other cell types. This protein has been shown to be of predictive value in several types of cancers (47) and to be specifically upregulated in primary cultures from giant cell tumors of bone and desmoplastic fibroma samples (48). The CD44 residues implied in OPN binding involve the region containing amino acids 121 to 140 (47). The CD44/OPN interaction (32) supports tumor progression through the activation of the phosphatidylinositol 3-kinase/Akt signaling pathway (49), increasing consequently cell survival. Several cells, such as MDA-MB-435 or LCC15-MB among other breast cancer cell lines produce increased levels of this protein (50), which provide them with a growth advantage. HIF‐2α (Hypoxia-Inducible Factor 2α) expression is also regulated by CD44‐OPN interaction through a CBP/p300‐dependent mechanism (mediated by CD44-ICD) and it has been demonstrated to promote stem-like properties and tumoral aggressiveness in glioma cells (51).

#### 2.2.3 Proteoglycans

Several proteoglycans are being shown to interact with CD44. For example, serglycin a proteoglycan characterized by repeats of Serine-Glycine dipeptide, is primarily expressed in hematopoietic tissue (52). However, this proteoglycan can be secreted by many cancer cells (e.g., nasopharyngeal carcinoma cells, myeloma cells, non-small cell lung cancers) (53, 54). CD44-Serglycin interaction maintains tumoral cell stemness through CD44 upregulation (as part of a positive feedback loop) mediated by MAPK/β-catenin pathway activation in nasopharyngeal carcinoma cells (55). Another proteoglycan, the versican, which is secreted by tumor stromal fibroblasts and cancer cells, binds to HA and generates HA/versican aggregations through its N-terminal region that contains a G1 domain (56), composed of an immunoglobulin-like domain (57, 58). The macromolecular complexes CD44/HA/versican promoted the invasion events in ovarian cancer cells (59). Another proteoglycan, aggrecan, a major component of cartilage, interacts with CD44, mediates cell adhesion, and induces CD44 oligomerization, which may lead to the triggering of the phosphorylation of Src kinases, activation of Rho-like GTPases or NF-κB activation cascades (60).

#### 2.2.4 Fibronectin and Collagen

Fibronectin and collagens are abundantly present in the ECM and are mostly found in connective tissues. The interaction of fibronectin with CD44 facilitates the extravasation of cells and the adhesion processes, allowing cancer cells to adhere more efficiently to ECM within a metastatic microenvironment (61). On the other hand, collagens are the main components of the ECM which must be degraded so that tumor cells can colonize other tissues and organs. CD44 can upregulate serine protease and collagen-degrading enzymatic expression and its activity to achieve it (62) (see the paragraph below matrix metalloproteinases). Additionally, CD44 and type IV collagen interactions have been involved to participate in cell adhesion processes in colorectal carcinoma cell lines (KM-12c, CCL 188, and MIP-101) (63).

#### 2.2.5 Matrix Metalloproteinases (MMPs)

MMPs are a group of calcium-dependent zinc-containing endopeptidases, which are responsible for tissue remodeling and degradation of ECM components, including collagens, elastins, gelatin, matrix glycoproteins, and proteoglycans (64). Malignant cells produce high levels of these proteins, which allow them to degrade ECM components faster than normal cells (65). The tumor invasiveness promoted by MMP-9 seems to be mediated by the binding of the proteolytically active metalloprotease to the CD44 ectodomain (66). Alternatively, the migration in rounded-amoeboid cells is supported through a non-catalytic mechanism based on actomyosin contractility modifications *via* CD44-MMP9 interaction (65). The metalloproteinase MT1-MMP, also known as MMP14, colocalizes with CD44 forming a complex through the hemopexin-like (PEX) domain in the MT1-MMP and the CD44-ICD, whose interaction is indispensable for degrading the extracellular matrix barrier during cancer invasion and is also involved in the CD44 ectodomain cleavage (see below) (67, 68).

### 2.3 Signaling Pathways Activated Through CD44

CD44-dependent signalling pathway activation takes place through two different mechanisms: i) the interaction between adaptor molecules and the intracellular domain (CD44-ICD) (28, 37) and ii) through the direct translocation of the CD44-ICD to the nucleus (37, 69, 70).

**Figure 3:**
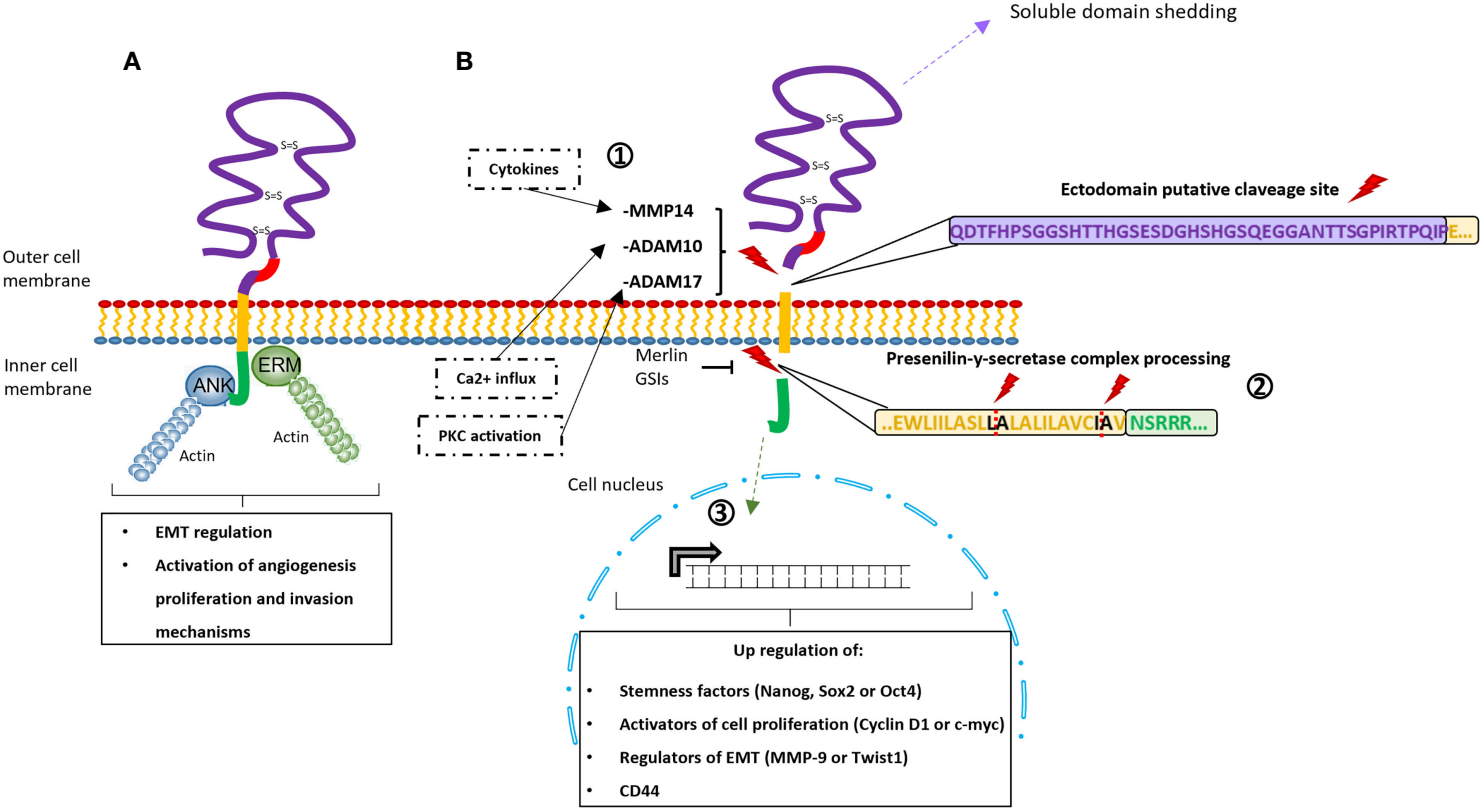
Schematic representation of the signaling pathways activated through CD44. **(A)** Interaction between adaptor molecules Ankyrin (blue) and Ezrin-Radixin-Moesin (green) with the intracellular domain CD44-ICD. This interaction triggers modifications in the cytoskeletal disposition and the activation of specific pathways (e.g., c-Met activation, Ras-MAPK cascade, Snail/β-catenin translocation, or PI3K-AKT pathway activation). As consequence, this *via* is involved in epithelial‐mesenchymal transition mechanisms (EMT) and the activation of angiogenesis, proliferation, and invasion mechanisms. **(B)** Schematic representations of CD44-ICD signaling via, including the sequential proteolytic cleavage of the CD44 protein. 1) The ectodomain shedding is induced by PKC, Ca2+ influx or cytokines that activate the MMPs (ADAM17, ADAM10 and MMP14, respectively). 2) The presenilin-y-secretase complex is activated and processes the ICD through the specific cleavage sites in the transmembrane domain (residues L-A and residues I-A). GSIs (γ-secretase inhibitors) and the merlin protein can inhibit this second proteolytic processing. 3) The ICD is released and translocated to the nucleus upregulating stemness factors, activators of cell proliferation, epithelial‐mesenchymal transition mechanisms (EMT) regulators and CD44 itself.

i) The CD44-ICD domain lacks kinase activity but it can interact with adaptor molecules like ankyrin and ERM (ezrin-radixin-moesin protein family) (**Figure 3**), which link CD44 to the actin‐cytoskeleton network. These interactions trigger changes in the tumor cell cytoskeletal architecture and cell signaling (e.g., c-Met activation, Ras-MAPK cascade, Snail/β-catenin translocation, or PI3K-AKT pathway activation), playing a role in the regulation of epithelial‐mesenchymal transition mechanisms (EMT) and the activation of angiogenesis, proliferation, and invasion mechanisms (70, 71).

ii) On the other hand, CD44-ICD can translocate to the cell nucleus thanks to the transportin1-specific nuclear localization signal (residues DRKPS) present at its N-terminal region and there (**Figure 3**) (71, 72). At the nucleus, CD44-ICD regulates the expression of some genes through the formation of different complexes with CBP/p300, Runx2, or Stat3 (42, 70, 73–76). Among the genes that have been shown to be regulated by CD44-ICD are some stemness factors (e.g., Nanog, Sox2, or Oct4), activators of cell proliferation (e.g. Cyclin D1 or c‐myc), regulators of EMT (e.g., MMP‐9 or Twist1) or CD44 itself (51, 69, 70, 73, 74, 76, 77).

The translocation of CD44-ICD to the nucleus is preceded by a sequential cleavage (**Figure 3**) of the CD44 protein (69). The ectodomain cleavage produced between the variable region and the transmembrane domain could be activated by three different pathways; i) the protein kinase C (PKC) which activates the ADAM Metallopeptidase Domain 17 (ADAM17); ii) the calcium influx which activates the ADAM10; iii) the interleukin 1, TGFβ1, or interferon γ which induces the expression of MT1-MMP (MMP14) activating proMMP-2 (78, 79), and promoting cell migration and invasiveness (80–82). Subsequently, a mechanism called regulated intramembrane proteolysis (RIP), which is driven by the presenilin‐γ‐secretases is activated. The presenilin‐γ‐secretase complex, constituted by presenilin 1 and presenilin 2 γ‐secretases, recognizes the proteolytic cleavage sites at the CD44 transmembrane domain (residues Ala278-Leu279 and Ile287-Ala288) and detaches the intracellular domain from the rest of the protein (83), releasing the CD44-ICD domain to the cytoplasm (70, 73, 84). Interestingly, this sequential proteolytic cleavage can be promoted by CD44-ligand interactions (26, 35, 79, 83, 85), while the CD44-ICD releasing can be inhibited by γ-secretase inhibitors (GSI) (69, 77) or by merlin protein. Merlin recognizes the same motif recognized by ERM (71) and can provide a tumor suppressor state by blocking the cleavage site (46).

## 3 MSCs, CSCs, AND DIFFERENTIATED TISSUES: CD44 EXPRESSION AND INFLUENCE DURING CELL DIFFERENTIATION AND SARCOMAGENESIS

Sarcomas are thought to arise from mesenchymal stem cells (MSCs) or derived progenitors, from which normal differentiated cells are also produced (for example adipo-, chondro-, myogenic, and osteocytic cells) (86). Therefore, the study of the similarities and differences among different grades of cell differentiation, that is, mesenchymal stem cells, sarcoma cells, and normal differentiated cells, is important to understand the role of CD44 in sarcomas.

### 3.1 CD44 Expression in MSCs, Sarcoma (CSCs), and Differentiated Tissues

**Figure 4:**
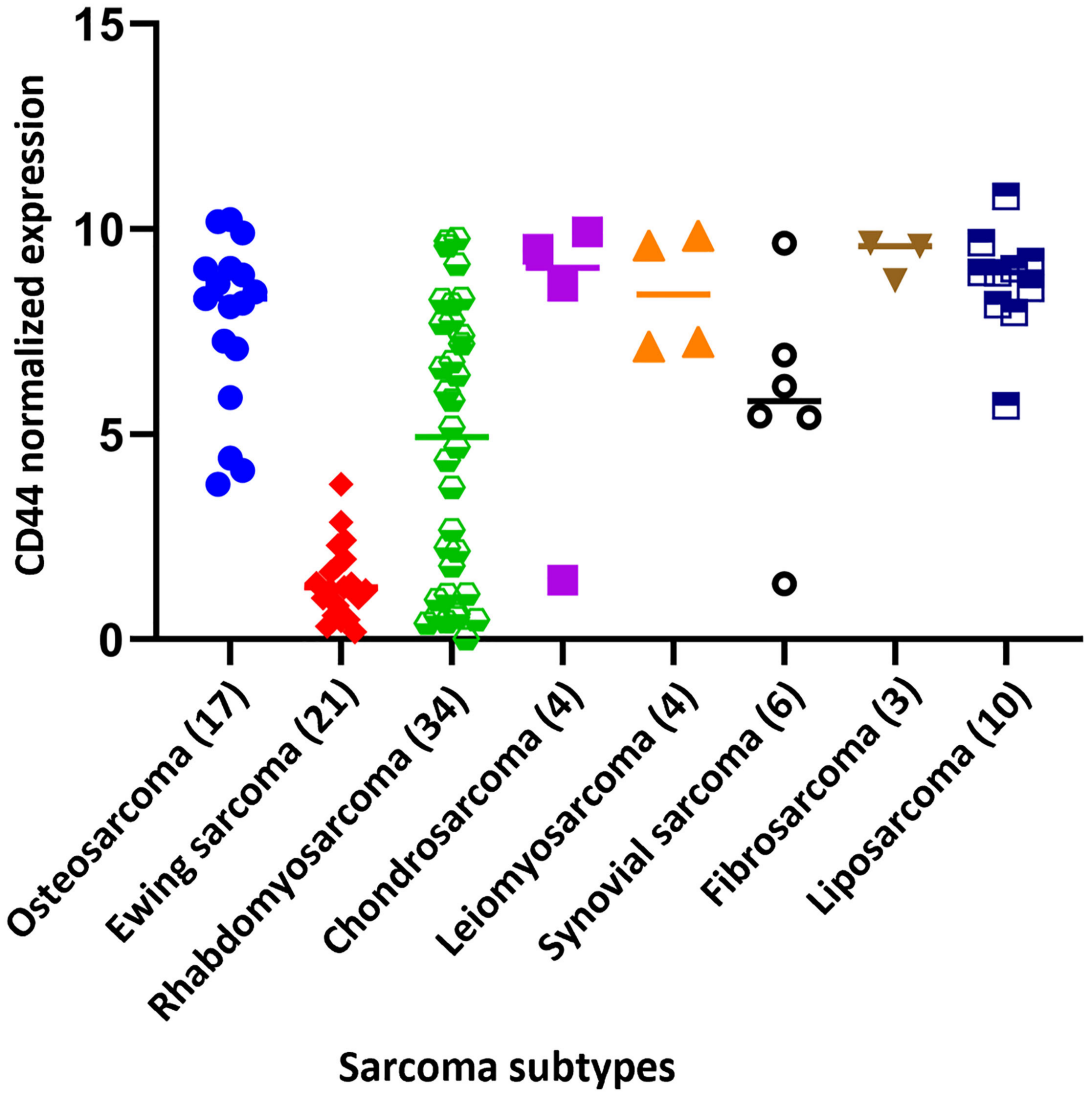
CD44 normalized expression of the different cell line sarcoma subtypes. Data extracted from DEPMAP Portal (https://depmap.org/portal/). The number of cell lines in each sarcoma subtype is indicated in brackets.

MSCs presents different surface markers including STRO-1, CD166, CD146, CD106, CD105, CD90, CD73, CD54, CD44, CD34, CD29 and CD13 (87, 88). CD44 shows high expression in MSCs as well as in sarcomas (89–92). Interestingly, CD44 has been shown to be expressed in the subpopulation of cancer stem cells (CSCs), which can reconstitute the tumor mass. CSCs and MSCs share similar stem-like properties and features. These similitudes among the characteristics of MSC (stemness, healing, regeneration processes, and cell recruitment) and cancer stem cells CSCs (potential for tumor initiation, EMT, tumoral progression, and stemness) with increased CD44 expression have already been broadly described in different cancers (28, 70, 93–97). Concordantly, CD44 is expressed at high levels in most sarcomas (**Figure 4**). The available data from the public database (DEPMAP Portal, https://depmap.org/portal/) confirms that CD44 expression was elevated in all sarcoma cell lines available in the dataset, with the unique notable exception of Ewing sarcoma, which expresses low levels of CD44 in comparison to the rest of sarcoma cell lines. Controversially, Skubitz et al., performed a CSCs characterization of stem cell markers in 31 soft tissue sarcoma from patients and determined that neither CD44, ALDH1, or CD133 were useful for sarcoma (CSCs) identification in these samples (98).

On the other hand, the CD44 protein is detected in the most differentiated tissues according to the Human Protein Atlas database (https://www.proteinatlas.org/). In consequence, CD44 shows a reduced tissue specificity due to its ubiquitous nature. Despite its ubiquity, CD44 is predominantly found in epithelial cells, bone marrow, and lymphoid tissue. Mesenchymal-derived tissues such as chondrocytes and bone cells have been demonstrated to express CD44 and modulate its expression in specific biological processes (cell-cell and/or cell-matrix attachment, morphological changes observed from osteoblasts to osteocytes, osteoclastogenesis, regulatory function in chondrocytes) (99–101). Additionally, CD44 seems to be involved in the processes of MSCs differentiation (101–105). Interestingly, CD44 expression was suggested to be reduced during the process of differentiation from MSCs to differentiated mature cells (103, 106). Thus, Huynh et al., monitored the process of chondrogenic differentiation from MSCs derived from bone marrow. They analysed the changes in the mRNA profile during differentiation and described several MSC markers, such as CD44, ENG, NT5E, or THY1, that progressively decreased during the differentiation process, while well-established chondrogenic markers (e.g., COL2A1, ACAN, COL9A1, COL11A1, COMP) were up regulated as the process of cell differentiation progressed (106). Moroes et al., studied the effect of CD90 and CD44 on differentiation processes induced in mesenchymal stem cells. The silencing of CD90 decreased the expression of CD44 which promoted the osteogenic and adipogenic differentiation of MSCs *in vitro*, thus associating the expression of high levels of CD44 and CD90 to the stemness state of MSCs (103, 107). Alternatively, the impairments in maturation processes involving high levels of CD44 were tested in neural differentiation experiments from MSCs and astrocytes precursor cells. Increased expression of CD44 inhibited oligodendrocytes differentiation and arrested the cells at the glial progenitor state. The *in vivo* research performed in a CD44 misexpression transgenic mouse model (CNP-CD44 mouse), supported that the CD44 overexpression blocked the oligodendrocyte maturation (108, 109). Complementary, Naruse et al., demonstrated that despite the widespread expression of CD44 in progenitor cells at early embryonic stages, the CD44 expression in adults was restricted to a specific mature subtype of cells (granule neurons). Thus, supporting the role of CD44 in the maintenance of stemness properties, but being able to promote differentiation of specific subtypes of neurons and astrocytes (110).

In summary, there is a relatively high expression level of CD44 in MSCs and sarcomas according to different studies and publicly available datasets. The differences in CD44 expression among the stem-like cells (MSCs and CSCs/sarcomas) and differentiated tissues suggest a down-regulation event of CD44 upon differentiation into mature cells, acquiring a new basal level of CD44, corresponding to its novel biological functions, which is susceptible of up/down-regulation.

### 3.2 CD44 and Tumor Microenvironment

The mesenchymal cell microenvironment has been demonstrated to be crucial for the development of multiple functions of mesenchymal stem cells (MSCs). This microenvironment provides components for cell regeneration and self‐renewal potential maintenance (111, 112). For instance, abundant quantities of HA are produced by MSCs to maintain their stemness (70). Injure healing processes and tissue regeneration are typical MSCs-driven processes, for which MSCs recruitment is mandatory, and CD44s-HA interaction seems to be a determinant mechanism to promote cell migration (113). Therefore, CD44 and the microenvironment play an important role in the processes performed by these cells and as consequence in their derived tumors.

Functionally, the high expression of CD44 benefits CSCs in several situations. CD44 works as a co-receptor for growth factors (CD44v3 co-receipts FGF and VEFG, while CD44v6 co-receipts EGF and HGF) and cytokines in the tumoral niche, potentiating receptor tyrosine kinase or CD44‐mediated signalling (37, 114) and triggering TGF-β up-regulation and the induction of Snail and Twist (115, 116). These two genes are considered key regulators of EMT and cancer progression (117–119). Snail has also been associated with cell cycle regulation, apoptosis evasion, cell adhesion, neuroendocrine differentiation, and chemoresistance (118–120). Twist is thought to promote metastasis, although the mechanisms implied are poorly understood (121, 122). Additionally, CD44-regulated transcription factors related to stem cell expression programs (Sox2, Klf4, Oct4, and Nanog), are commonly identified in MSCs and tumors (115), when CD44 has been knocked-down the expression of these markers (Oct4, Nanog, and Sox2) are notably reduced (123). Consequently, the transcriptional networks mediated by these factors are crucial for stemness properties observed during tissue development (70, 124, 125).

The hypoxic low-ROS (reactive oxygen species) environment is also associated with proliferation and the maintenance of self‐renewal capacities in cancer stem cells (CSCs) and solid tumors (126, 127). In this situation, CD44 has a specific function. HIF‐1α induced by hypoxia promotes glycolytic metabolism, angiogenesis (128), and CD44, CD44v6/v7/v8 expression (129). CD44 regulates ROS-mediated cytotoxicity in tumors through two different mechanisms (70). The interaction of CD44‐ICD-PKM2 (pyruvate kinase isoenzyme type M2) increases PKM2 phosphorylation (suppressing its activity) which promotes the activation of glycolytic metabolism, driving the CSCs to a pro-antioxidant status (130). Low PKM2 activity increases the levels of reduced NADPH which favors the regeneration of reduced glutathione GSH (131, 132). Alternatively, the interaction CD44-XCT (subunit of the cystine‐glutamate transporter Xc), promotes cystine uptake for the synthesis of GSH (133). As consequence, oxidative stress is controlled and reduced, providing a favorable environment for CSCs. The relevance of this mechanism has supposed its targeting through xCT inhibitors, like the sulfasalazine. This molecule has been tested in a dose-escalation clinical study with advanced gastric cancer patients, showing the inhibition of the proliferation in CD44v-positive cancer cells, both *in vitro* and *in vivo* (134).

## 4 Functional Characterization of CD44 in Sarcoma Cell Lines

Most of the studies carried out to understand the functionality of CD44 in cancer have been performed in epithelial tumors, although some relevant works focused specifically on sarcomas.

### 4.1 Chondrosarcoma

Some studies performed in chondrosarcoma cell lines have contributed to understanding the role of CD44 in these tumors and clarifying the signal transduction cascade triggered upon CD44 stimulation. Suzuki et al., reported that LMW-HA (molecular weight 3.5 kDa) can stimulate CD44 and subsequently, increase c-Met expression and c-Met phosphorylation in the chondrosarcoma cell line HCS-2/8 (45). In a similar study, using the same chondrosarcoma cell line, Kobayashi et al., demonstrated that the mRNA and protein of the urokinase-type plasminogen activator (uPA) and theurokinase-type plasminogen activator receptor (uPAR) were upregulated after CD44 stimulation with LMW-HA. Moreover, it was shown that CD44 stimulation with LMW-HA induced MAPK pathway activation, *via* phosphorylation of MEK1/2, ERK1/2, and c-Jun. Additionally, the signalling cascade derived from the interaction of HA with CD44 could be disrupted at different points; by using anti-CD44 antibodies, MAP kinase inhibitors, neutralizing anti-uPAR pAb, anti-catalytic anti-uPA mAb, or amiloride (135). These results provide a novel therapeutical alternative and suggest that there could exist an autocrine loop; CD44 stimulation - MAP kinase cascade activation (including c-Met) - uPA/uPAR overexpression, in chondrosarcoma cells that can boost their invasiveness.

### 4.2 Osteosarcoma

Gene silencing and CRISPR/Cas9-mediated knockout have been used to elucidate the role of CD44 in osteosarcoma pathogenesis, particularly in relation to resistance to treatment and metastasis formation. Knocking-out of CD44 using CRISPR/Cas9 system in the drug-resistant osteosarcoma cell lines KHOSR2 and U-2OSR2 (both resistant to Doxorubicin) showed significant inhibition of migration, invasion, proliferation, and resistance to doxorubicin (136). In another study, CRISPR/Cas9 was also used to knocking-out CD44 in the human osteosarcoma cell lines MNNG/HOS and 143B, both highly metastatic. This study showed the inhibition of cell proliferation and tumor-sphere formation in 3D cultures upon CD44 inactivation (137). CD44 inactivation, through a siRNA strategy, performed by Kong et al., in MG63 and U2OS cells showed a reduction in proliferation rate and inhibited the cell migration and the invasive capability (138). It was suggested that this effect could be a consequence of the downregulation of cathepsin S (a lysosomal cysteine protease of papain subfamily) upon CD44 knocking out. Concordantly, a similar study performed by Gvozdenovic et al., in 143B osteosarcoma cell line using an shRNA-silencing approach to downregulate CD44 expression, resulted in a decrease in the adhesion of osteosarcoma cells to HA, migration, and a diminished capacity to growth anchorage independent in soft agar. However, CD44 silencing favored the growth of the primary tumor and the appearance of pulmonary metastasis in an intratibial xenograft model (139), highlighting the existence of controversial results between *in vitro* and *in vivo* situations. Immunohistochemistry analysis of intratibial primary tumor and lung metastasis showed notably lower levels of merlin, a tumor suppressor protein, compared to the same cell lines in *in vitro* conditions. Therefore, this enhanced malignant phenotype *in vivo* could be associated with the impairment of the adhesion properties to ECM due to CD44 depletion or the reduced expression of merlin in *in vivo* conditions. The authors explained that since 143-B cells were obtained through Ki-Ras transformation (140), the enhanced Ras-driven metastatic behavior is evidenced because of merlin protein expression lacked *in vivo*. Although the mechanism involved in merlin downregulation in osteosarcoma cells has not been yet elucidated, a possible explanation could be deduced from studies carried out in breast cancer cells. In breast cancer cells, the phosphorylation of merlin which promotes its proteasomal degradation is a process initiated by OPN and mediated by the Akt pathway (141), thus suppressing completely the antitumoral activity of merlin. Interestingly, the CD44 expression in osteosarcoma CSCs has been determined to be affected by pimozide treatment (a STAT5 inhibitor). This drug reduced considerably the expression of CD44 among other stem cell markers (CD133, Oct-4, and ABCG2) in KHOS/NP and SJSA-1 cell lines and, in consequence, impaired the growth and stemness of CSCs (142). The available data from the depmap portal (https://depmap.org/portal/) showed some differences in CD44 expression among osteosarcoma cell lines. Thus, MG63, OS252, C396, and HS888T cell lines showed the highest levels of CD44 expression, while NOS1 and HSOS1 had the lowest levels.

In addition to the experimental strategies described above, the study of miRNAs and long non-coding mRNAs that can downregulate CD44 levels have been also useful to determine the role of CD44 in osteosarcoma pathogenesis. For example, ectopic expression of miR-34a represses the expression of CD44 in two osteosarcoma cell sublines (F5M2 with highly metastatic potential and F4 with a low metastatic potential), inhibiting their migration and invasive ability (143). Another miRNA, the miR-199a-3p that targets directly the 3′-UTR region of CD44, produces a reduction in the levels of CD44 mRNA. Interestingly, miR-199a-3p levels are notably reduced in osteosarcoma cells and tumors compared to normal osteoblast. The overexpression of miR-199a-3p in KHOS and U-2OS inhibited CD44 mRNA levels and significantly increased the sensitivity of these cells to doxorubicin (144). In other studies, *in vitro* knockdown of the oncogenic long non-coding RNA GAPLINC (Gastric adenocarcinoma predictive long intergenic non-coding) in HOS and Saos-2 osteosarcoma cell lines, reduced migration and invasion by inhibiting considerably CD44 expression, although it does not impair cell proliferation (145). In summary, these studies suggest that the CD44-miR-199a-3p axis and GAPLINC regulation are relevant regulators in the development of metastasis, tumor recurrence, and drug resistance in osteosarcoma cells mediated by CD44.

### 4.3 Ewing Sarcoma

Ewing sarcoma, an extremely rare sarcoma that arises mainly during childhood and adolescence, presents a notably reduced expression of CD44 compared to the rest of the sarcoma cell lines (**Figure 4**). The explanation for this difference could rely on the CD44 gene regulation, which contains an element recognized by the ETS-1 (v-ets erythroblastosis virus E26 oncogene homolog 1) family transcription factors. Ewing sarcoma is genetically characterized by a balanced chromosomal translocation, in which a member of the FET gene family (FUS, EWSR1, or TAF15) is fused with an ETS transcription factor (FLI1, ERG, ETV1, ETV4, or FEV, among others) (146). The EWSR1–FLI1 chimeric protein can recognize the domain on the CD44 promotor and regulates its expression. An assay performed in the A673 cell line with an shRNA silencing approach for EWSR1-FLI1 showed that EWSR1-FLI1 inhibition induces CD44, CD59, CD73, CD29, and CD54 surface antigen expression, among others (147). Therefore, the lower CD44 levels find in this rare cancer are due to the high basal expression levels of the EWSR1-ETS1. This phenomenon, regarding the downregulation of CD44 in comparison to other sarcomas, could be determinant for the tumor growth, dissemination, and drug resistance in this sarcoma subtype. Thus, further research is required to define the role of CD44 in Ewing sarcoma.

Beyond this observation, the role of CD44 in the progression, metastasis, or drug resistance of Ewing sarcoma remains unclear. Recently, Paulis et al., showed that CD44 was involved in a phenomenon known as ‘vasculogenic mimicry’, defined as the ability of some cells to transdifferentiate into cells with endothelial characteristics and constitute vasculogenic networks independent of angiogenesis, which is associated with tumor aggressiveness and poor prognosis. The authors used two derived Ewing sarcoma cell lines (EW7 and SIM.EW27) and two breast cancer cell lines (MDA-MB-231 and MCF-7) to study key molecules involved in the vasculogenic mimicry. The authors concluded that CD44/c-Met signalling cascade, highly overexpressed in the aggressive cell lines (EW7 and MDA-MB-231), is crucial for vasculogenic mimicry (148). Overexpression of CD44 (CD44s and CD44v6 isoform) in the EW7 and MDA-MB-231 cell lines (highly metastatic) compared to the low-invasive cell lines (SIM.EW27 and MCF-7) was related to increased aggressiveness and to the promotion of the formation of vasculogenic structures *in vitro*. Accordingly, knocking-out CD44 by siRNA reduced the CD44 RNA level and the protein expression to approximately 40% in EW7 cells. As result, the migration capacity was markedly reduced, as well as the vascular-like networks. Tumor samples from patients analyzed in this study also supported the association among vasculogenic structures and ‘blood lakes’ formation and CD44 expression. CD44 was detected by immunochemistry in 14 out of 15 Ewing sarcoma tissues whose score was positive for the presence of blood lakes (149).

### 4.4 Fibrosarcoma

Several studies involving fibrosarcoma cell lines have been performed to elucidate the role of the receptors for hyaluronic acid-mediated motility (RHAMM, another HA receptor) and HA interactions in fibrosarcoma. Kouvidi et al., determined the effect of HA/RHAMM signalling on the ability of HT1080 fibrosarcoma cell line to adhere to fibronectin. On one hand, the adhesion properties of HT1080 cells significantly increased (p ≤ 0.01) with LMW-HA treatment, while HMW-HA inhibited them. On the other hand, the HT1080 RHAMM-deficient cells had a diminished adherence capacity compared with control cells. Additionally, *in vitro* experiments established that the activation of FAK and ERK1/2 signalling pathways, triggered through the RHAMM/HA interaction, regulated cell adhesion properties in HT1080 fibrosarcoma cells (150). Hatano et al., reported that, in *in vitro* conditions, RHAMM interacted with ERK (as previously explained), increasing the proliferative ability of fibroma cells. However, this process involved the interaction of CD44 with the epidermal growth factor receptor (EGFR) (151). Therefore, the axis CD44/RHAMM seems to mediate proliferation and metastatic dissemination due to the modification of adhesiveness to ECM components.

The specific contribution of CD44 in metastatic dissemination was studied in murine models (152). Culp et al., overexpressed the human CD44s (standard isoform) on the nonmetastatic cell lines: sis-transformed Balb/c 3T3 cells and ras-revertant IIIA4 cells. Afterward, the cells were implanted in athymic nude mice. The result of the experiment demonstrated that the increased expression of human CD44s in the mice model promoted micrometastasis events of the lungs (152).

The functional studies performed in different sarcomas demonstrate that CD44 plays a relevant role in sarcomagenesis and tumor progression. Ligand-dependent processes, proliferation events, migration/adhesion capacities, or drug resistance were shown to be regulated through specific mechanisms in which CD44, ECM components, and microenvironment have a direct or indirect effect. Although several studies have shown a relevant role of CD44 in a reduced number of sarcomas, there is a clear lack of studies in other types of sarcomas such as myxofibrosarcoma, synovial sarcoma, or rhabdomyosarcoma, among others. This highlights the existence of a poorly developed field of research that needs to be completed to define in detail the role that CD44 plays in these types of cancer.

## 5 Prognostic Value of CD44 Expression in Sarcomas

Several studies have analyzed the prognostic value of CD44 expression in sarcomas (**Table 1**). Given that sarcomas are rare cancers, and therefore it may be difficult to accumulate a relevant number of cases, several of these studies have been carried out with a series of patients consisting of various tumor types. Henderson et al., analyzed the expression of CD44 by immunohistochemistry in a series of 23 soft tissue sarcomas (8 liposarcomas, 4 myxofibrosarcoma, 4 undifferentiated pleiomorphic sarcomas, 1 alveolar soft parts sarcoma, 1 extraskeletal mesenchymal chondrosarcoma, 1 leiomyosarcoma, 1 malignant peripheral nerve sheath tumor, 1 extraskeletal osteosarcoma, 1 synovial sarcoma, and 1 primitive neuro-ectodermal tumor. Their results suggest that increased CD44 expression was associated with a worse disease specific survival (p=0.056, univariant analysis). In the same article, the authors analyzed by multivariate analysis 74 cases of soft tissue sarcoma from The Cancer Genome Atlas program (TCGA) that included clinical and genomic data (https://www.cancer.gov/about-nci/organization/ccg/research/structural-genomics/tcga). It was shown that CD44 copy number variations predicted worse disease specific survival (p=0.007). Thus, the authors concluded that increased expression of CD44 was correlated with worse outcomes in soft tissue sarcomas (153). In another study, Kahara et al., analyzed by immunohistochemistry the expression of several CD44 isoforms in a series of 47 sarcomas (18 malignant fibrous histiocytomas, 13 synovial sarcomas, 7 malignant schwannomas, and 9 liposarcomas). Isoforms CD44v3, v4, v5, v6, v7, and v9 were frequently detected, whereas CD44v10 was not expressed in any of the samples analyzed. CD44v6 isoform was commonly detected in high-grade tumors and both CD44v6 and v9 expression correlated negatively with metastasis-free survival (154). Kebudi et al., also studied the relevance of CD44 levels in serum (measured by ELISA), in the context of pediatric sarcomas (18 rhabdomyosarcomas, 22 Ewing sarcoma, and 15 osteosarcomas). In this study, the differences observed between the serum levels of CD44 in children with sarcoma and healthy controls were not significant (p>0.05), as result, a non-diagnostic or prognosis value was established (155).

**Table 1:** Summary of sarcoma subtype studies analyzing CD44 expression alone or in combination with other biomarkers in patient samples.

Sarcoma subtype	Study	Number of samples	CD44 isoform detected	Detection technique	Additional markers tested	Clinical conclusion
Myxofibrosarcoma	Matuschek et al.	34	Increased CD44s	Quantitative PCR	–	Improved clinical outcome
			Low CD44v6			
	Tsuchie et al.	44	Increased CD44s	Inmunohistochemical	–	Poor event free survival and local recurrence in patients with lung metastasis
Synovial Sarcoma	Sneath et al.	56	CD44 expression	Inmunohistochemical	–	No correlation with prognosis
	Zhou et al.	20	CD44 expression	Inmunohistochemical	CD133, CD29, Nestin and ALDH1	CD44 was not correlated with prognosis, but ALDH1 positive cases showed a poorer prognosis
Rhabdomyosarcoma	Saxon et al.	12	CD44 expression	Inmunohistochemical	ECM proteins (laminin, fibronectin, tenascin and thrombospondin)	No correlation among the markers tested with metastasis or with the level of tumor differentiation
	Humphrey et al.	28	CD44 expression	Inmunohistochemical	–	CD44 positive patients presented improved outcome
Chondrosarcoma	Heyse et al.	30	CD44 overexpression	Inmunohistochemical	–	Correlation with tumor grading, metastatic potential, and survival
	Roxeman et al.	16	CD44 expression	Inmunohistochemical	ECM components, growth factors, p53, among others	Preferential CD44 splicing: CD44+/CD44v3− in the chondrogenic component of dedifferentiated peripheral chondrosarcoma and CD44−/CD44v3+ in secondary peripheral chondrosarcomas
Osteosarcoma	Boldrini et al.	34	CD44 expression	Inmunohistochemical	Ezrina	No correlation was established
	Gvozdenovic et al.	53	CD44 expression	Inmunohistochemical	–	Failed as an independent predictor
	Liu et al.	329	CD44 expression	Meta-analysis	–	CD44 expression did not correlate with overall survival or metastasis
	Xiao et al.	96	CD44 expression	Inmunohistochemical	–	Useful biomarker to predict chemoresistance. Supported by functional studies
	Liu et al.	548	CD44 expression	Meta-analysis	–	Useful in the prediction of poor survival and metastatic potential
	Kuryu et al.	44	CD44s and CD44v3, v4, v5, v6, v7, v9, and v10	Inmunohistochemical	–	CD44v6 was correlated to patient prognosis
	Deng et al.	110	CD44v6	Inmunohistochemical	CDH11 and β-catenin	CD44V6, CDH11, and β-catenin were associated with overall survival
	Gao et al.	114	CD44 expression	Inmunohistochemical	–	Poor outcome and drug response
	Kim et al.	59	CD44 expression	Inmunohistochemical	IGF1R and ABCG2	CD44 and ABCG2 can be used in combination with IGF1R as prognosis and efficient treatment factors
	Zhang et al.	486	CD44v6	Meta-analysis	–	CD44v6 over-expression correlates with poor outcome and metastasis
	Zhang et al.	463	CD44v6	Meta-analysis	–	CD44v6 could act as a diagnostic marker for osteosarcoma

### 5.1 Studies Performed on Specific Sarcoma Subtypes

#### 5.1.1 Myxofibrosarcoma

Myxofibrosarcoma is one of the most common and aggressive soft tissue sarcomas, commonly developed in the limbs. A multivariate study focused on the analysis of CD44 isoforms in 34 adult patients with myxofibrosarcoma showed that increased expression of CD44s and reduced expression of CD44v6 isoform correlated significantly with an improved outcome (p<0.05 and p<0.02, respectively) (156). Additionally, a recent multivariate analysis designed to identify possible relationships between CD44s expression and clinic-pathological factors carried out in 44 retrospectively enrolled myxofibrosarcoma patients, showed that increased expression of CD44s correlated negatively with event-free survival and local recurrence. Additionally, in the subgroup of patients with distant metastasis, the CD44 expression was significantly elevated in patients with lung metastasis compared to patients with lymph node metastasis (p=0.044) (157).

#### 5.1.2 Synovial Sarcoma

Synovial sarcoma is an aggressive soft tissue sarcoma, whose initiation and progression rely on the fusion gene SYT-SSX (158). This tumor has a low incidence, and it is predominantly detected in the lower extremities of young adults (8). This tumor is formed by mesenchymal spindle cells, but it is not related to the synovial membrane. Studies of immunohistochemistry determined that CD44 expression did not correlate with prognosis (survival, local recurrence, or metastasis) in synovial sarcoma (159). The uselessness of CD44 levels in synovial sarcoma prognosis has also been supported by Zhou et al., where stem cell-associated markers (CD133, CD29, CD44, nestin) and ALDH1 were characterized immunohistochemically in 20 synovial sarcomas. No relationship was settled down between these markers and clinical parameters (age, gender, sites, tumor size, histological type, tumor stage, and distant metastases). However, this study confirmed that ALDH1 positive synovial sarcomas had a significantly poorer prognosis compared to ALDH1 negative synovial sarcomas (160), providing an alternative prognosis marker to CD44.

#### 5.1.3 Rhabdomyosarcoma

Rhabdomyosarcoma is a malignant soft tissue tumor that arises from muscle cells. It commonly arises during childhood. There are two main types of rhabdomyosarcomas that differ in their molecular and clinical characteristics. Alveolar rhabdomyosarcomas are characterized by the presence of reciprocal translocations that result in chimeric proteins that fusion the PAX3 or PAX7 genes with the transcription factor FOXO1. By contrast, embryonal rhabdomyosarcomas are characterized by chromosomal losses and gains (161). Humphrey et al., analyzed by immunohistochemistry, a series of 28 rhabdomyosarcomas (8 alveolar and 20 embryonal) and showed that CD44 positive patients had improved outcomes compared with CD44 negative tumors (p=0.001, Fisher test) (162). By contrast, a study performed by Saxon et al., in 12 pediatric rhabdomyosarcoma tumor-derived cells (five alveolars, six embryonal, and one botryoid subtype) reported no association between the expression of ECM proteins (laminin, fibronectin, thrombospondin, tenascin) and CD44 with metastatic events at clinical presentation (163).

#### 5.1.4 Chondrosarcoma

Chondrosarcoma is a type of sarcoma that develops from chondrocyte progenitors, primarily affecting the cartilage of the femur, arm, pelvis, or knee. Heyse et al., determined that the levels of CD44s increased with the malignant grade of the tumor and that the overexpression of CD44 correlated with metastatic potential and survival (164). This study included 22 conventional chondrosarcomas, two dedifferentiated chondrosarcomas, two extraskeletal chondrosarcomas, and one periostal, mesenchymal, clear cell, and myxoid chondrosarcoma each. In another study, Rozeman et al., analyzed sixteen chondrosarcomas by immunohistochemistry for CD44, different ECM components, and growth factors. The study suggested the existence of a differential pattern of CD44 isoform. Thus, CD44 positive and CD44v3 negative expressions were found in the chondrogenic component of dedifferentiated peripheral chondrosarcoma, whereas in secondary peripheral chondrosarcomas was detected the expression of CD44 negative− and CD44v3 positive isoforms (165). Dedifferentiated peripheral chondrosarcoma is a rare subtype of chondrosarcoma arising superimposed on the cartilage cap of a pre-existing osteochondroma (8). These findings suggest that different CD44 isoforms can be associated with different grades of chondrogenic differentiation in chondrosarcomas. No data about the correlation of clinical parameters with CD44 expression were present in this study.

#### 5.1.5 Osteosarcoma

Osteosarcoma is one of the most common sarcomas observed during childhood and adolescence (8). Approximately 30% of patients develop pulmonary metastasis (166). Some parameters well-established that affect the patient outcome are the anatomic location of the primary tumor, tumor size, dissemination processes, and response to induction chemotherapy (167). These tumors are the sarcomas in which more studies on CD44 have been carried out although the role of CD44 as a prognostic marker in osteosarcoma patients is controversial. Some studies, including one meta-analysis of 329 patients from six different studies (168), conclude that there is no significant association between prognostic or metastatic recurrence with CD44 expression levels (168–170). However, recent studies, including other meta-analyses of 548 osteosarcoma patients from nine studies, have concluded that CD44 expression may predict survival, metastasis recurrence, and drug resistance (136, 137). This agrees with *in vitro* studies carried out in different osteosarcoma cell lines (KHOS doxorubicin resistant, U2-OS doxorubicin resistant, MNNG/HOS, and 143B) that demonstrated that CD44 promotes cell migration, cell invasiveness, and drug resistance (136, 137, 139, 170).

Other studies focused on osteosarcoma have revealed that overexpression of CD44v6 (171, 172), CD133 (173), CD133/CD44 combination (174), CDH11 (172), β-catenin (172), GAPLINC (145), CD44-miR-199a-3p axis (144) and the combination CD44/IGF1R/ABCG2 (175) could provide valuable information as prognostic markers in this sarcoma. Supporting the role of the CD44v6 marker in osteosarcoma, two meta-analyses (both with more than 460 osteosarcoma cases) focused on the prognostic utility of this specific CD44 isoform revealed that its overexpression is associated with the overall survival rate and the occurrence of metastatic events and could be used as a complementary diagnostic marker in osteosarcoma (176, 177). However, the authors explained that the diagnostic utility of CD44v6 presented some limitations due to the low number of articles involved in the meta-analysis and the heterogeneity among them (because of the cut-off values, control groups, assay kits, and other aspects). Interestingly, the CD44v6 variant is one of the CD44 isoforms that more frequently correlates with tumor progression in other malignancies. For example, poor prognosis, presence of metastasis, or disease progression have been correlated with CD44v6 expression changes (alone or in combination with other isoforms) in gastric carcinoma (178, 179), non-small cell lung carcinoma (180, 181), hepatocarcinoma (182), acute myeloid leukemia (183), non-Hodgkin’s lymphoma (184, 185), pancreatic adenocarcinoma (186), primary pancreatic cancer (187) and uterine cervical carcinoma (188). Therefore, the CD44v6 variant seems to play a critical role in disease progression and could provide useful prognostic information in osteosarcoma, among other malignancies.

The combination of CD44 with other surface markers can provide additional information about the malignant potential of osteosarcoma cells. For example, in nude mice intratibial xenograft model performed with Saos-2 osteosarcoma cell line, CD133+/CD44+ cells were potentially more metastatic (174). A multivariate analysis performed on 90 osteosarcomas and 20 osteochondromas, indicated the clinical stage, metastasis status, and the relationship between the expression of CD44V6, CDH11, and β-catenin correlated with improved outcome (172). GAPLINC showed an indirect regulation of CD44 due to both mRNAs are negatively regulated by the same microRNA, the miR211-3p. GAPLINC overexpression in osteosarcoma was shown to correlate with advanced Enneking stage, distant metastasis, and poor outcome (145), providing an additional novel biomarker. The expression of CD44/IGF1R/ABCG2, tumoral markers related to prognosis and drug resistance, have also been analyzed, alone and in combination, in order to obtain information about their contribution to the patient prognosis. Thus, the Insulin Growth Factor 1 receptor (IGFR1) has been shown to be a prognostic factor for metastasis in osteosarcoma (189). The analysis of the expression pattern in 59 osteosarcomas showed that IGF1R expression is highly correlated with ABCG2 expression and with CD44 expression in osteosarcoma patients under 10 years old (175).

## 6 Therapeutic Approaches Targeting CD44

CD44 can be considered a suitable therapeutic target in many malignancies by blocking the activation of the signalling pathways in which this protein is involved, or by using its overexpression to drive, specifically, the pharmacological treatment. In fact, several strategies targeting CD44 have already been developed which include small molecular inhibitors, peptides, aptamers, blocking antibodies, secretase inhibitors, drug delivery systems, CD44 decoys, and HA oligomers (**Table 2**).

**Table 2:** Summary of therapeutic approaches targeting CD44.

Pharmacological approach	Agent	Mechanism of action	Stage of development	Refs
Small molecular inhibitors	Silibinin	Inhibits the activity of the CD44 promoter reducing its expression.	Preclinically tested in prostate, pancreatic, and breast cancer cells	(190, 191)
	Zerumbone	Suppresses EGF-dependent CD44 expression through inhibition of the STAT3 pathway.	Preclinically tested in breast cancer cells.	(192)
	Curcumin and epigallocatechin gallate	Reduces CD44 expression through the inhibition of the STAT3 pathway (blocks the STAT3 phosphorylation).	Alternative approach like Zerumbone. Not tested yet.	(193)
GSI (γ-secretase inhibitors)	PF-3084014 (Nirogacestat)	Blocks CD44-ICD releasing and subsequently interfere with CD44-ICD-dependent functions.	Phase II. Clinical benefit in patients with refractory, progressive desmoid tumors who receive long-term treatment (NCT01981551).	(194)
	LY-450139 (Semagacestat)		Phase I. Clinical activity in heavily pretreated patients with breast cancer and leiomyosarcoma (NCT01695005).	(195)
	BMS-906024		Preclinically tested. Decreases cell proliferation in *in vivo* studies using cell line- and patient-derived lung adenocarcinoma xenografts.	(196)
Antibodies conjugated with anti-tumor drugs	RO5429083 (Roche)	Immunoconjugates antibody that binds to the constant region of the extracellular domain, favoring the antitumoral drug uptake.	Phase I. Tested in patients with metastatic and/or locally advanced CD44-expressing malignant solid tumors (NCT01358903).	(197, 198)
	U36 indium-111	Monoclonal antibody labeled with indium-111 that targets CD44 and had been suggested the possible use in the detection of this cancer.	Preclinically characterized in head and neck carcinoma xenografts mice models expressing CD44v6 isoform.	(199)
	Bivatuzumab mertansine	Humanized monoclonal antibody against CD44v6 and a cytotoxic agent (mertansine).	Phase I. Tested in head and neck carcinoma patients with variable therapeutic response and a severe skin toxicity	(200, 201)
Antibodies blocking CD44	H4C4	Decreases the capacity of self-renewal and tumor initiation through STAT3 signaling inhibition and the downregulation of the stem cell self-renewal gene Nanog.	Preclinically tested. Reduces tumor growth, metastasis, and post-radiation tumor recurrence in human pancreatic mice xenografts.	(202)
	IM7	Inhibits HA-CD44 mediated signaling in human umbilical vein endothelial cells.	Preclinically tested. Additionally, decreases cell migration and invasion capacities in breast cancer cell lines.	(203, 204)
	KM201		Preclinically tested in human umbilical vein endothelial cells.	(203)
Peptides and aptamers	PEP-1	Reduces CD44 expression levels in mice models for gastric cancer.	Preclinically tested.	(205)
	PCK3145	Seems to interfere with the tyrosine kinase activity associated with the VEGF signalling axis in endothelial cells inhibiting angiogenesis processes.	Preclinically tested. Demonstrated to reduce bone metastases and prostate tumor growth tumor in rats inoculated with MAT-Ly-Lu-B-2 cell line.	(206–208)
	Apt1 (RNA aptamer)	Used to functionalize the surface of PEGylated liposomes, increasing cellular uptake of CD44 positive cell lines.	Preclinically tested in cell lines: A549 (lung cancer) and MDA-MB-231 (breast cancer).	(209)
	CD44-EpCAM (double-stranded RNA aptamer)	Blocks simultaneously CD44 and EpCAM, reducing tumor progression and promoting apoptosis.	Preclinically tested. *In vitro* and *in vivo* xenograft models of ovarian tumor cells (OVCAR8).	(210)
Ligand chemotherapy delivery systems	Chol-SS-mPEG/HA-L	HA-coated redox responsive liposome, whose cytoplasmic drug release system is triggered by GSH.	Preclinically tested in xenograft models of osteosarcoma. The results showed: a reduction of tumor growth and increased animal survival.	(211)
	HA-LsDOX	Promotes the sulfhydration and ubiquitination of proteins and activates the pro-apoptotic CHOP-mediated signaling.		(212)
	ALN–HA–SS–L–L/DOX	Equipped with bone- and CD44-dual-targeting and redox cleavage characteristics, its efficacy seems to increase with the coadministration of internalizing RGD.		(213)
	HA-es-ZnPP	Photodynamic therapy based on a hyaluronan conjugated zinc protoporphyrin *via* an ester bond, with a tumor environment-responsive mechanism.	Preclinically tested *in vitro* and in a mouse sarcoma S180 solid tumor model, demonstrating prolonged circulation time, enhanced cell permeability and retention and anticancer effect.	(214)
	ALN-HA-C18/curcumin	Dual-targeting delivery therapy combining the active bone accumulating ability and the curcumin inhibition effect on CD44 expression.	Tested preclinically in MG-63 cells and in *in vivo* model. The results showed a reduction of tumor growth in osteosarcoma mouse model.	(215)
Hyaluronic Acid oligomers	HA-mers	Bind CD44, competing by and displacing the biological HA polymer and can inhibit HA synthesis.	Preclinically tested. Promoted apoptosis and reduced both cell viability, cell proliferation, cell motility, and decreased the retention of endogenous HA in murine (LM-8) and human (MG-63) osteosarcoma cells. Its intratumoral injection in xenograft models suppressed dissemination events in the lung. A similar effect has been observed in other cancers such as melanoma, carcinoma, or glioma.	(94, 216, 217)

### 6.1 Inhibition of CD44 Expression

Since CD44 is expressed at high levels in most types of sarcoma, inhibition of CD44 expression could have therapeutic usefulness. In this sense, several compounds have been shown to regulate the expression of CD44, both directly and indirectly (218). Silibinin, a flavonolignan composed of two diastereomers (silibinin A and B), strongly inhibited the activity of the CD44 promoter in the prostate (PC-3M) and pancreatic cancer cell lines (BxPC-3 and PANC-1) (190, 191). Zerumbone, a sesquiterpenoid and cyclic ketone, suppressed EGF-dependent CD44 expression through inhibition of the STAT3 pathway in breast cancer cell lines (SKBR3 and MDA-MB468) (192). Additionally, the inhibition of the STAT3 pathway (through the selective blocking of STAT3 phosphorylation) was obtained with the combination of curcumin and epigallocatechin gallate in a pharmacological treatment, which reduced the CD44+ population of CSCs in MDA-MB-231 and MCF7 breast cancer cell lines (193). Therefore, the use of Sibilin, Zerumbone, or curcumin/epigallocatechin notably reduced the population of CD44+ tumor cells (breast cancer, pancreatic cancer, and prostate cancer), suggesting a novel approach for the treatment of sarcomas.

### 6.2 Inhibition of γ-Secretases

As described above, CD44-ICD is released upon cleavage of CD44 by γ‐secretases and, subsequently, it is translocated to the nucleus where upregulates the expression of specific target genes implied in stemness, migration/invasiveness, or cell proliferation. The cleavage mechanism requires, inexorably, the activity of the presenilin‐γ‐secretases (aspartyl proteases). Thus, direct inhibition of these enzymes with γ-secretase inhibitors (GSIs) is a particularly interesting approach to block CD44-mediated signalling because these inhibitors can block CD44-ICD release and, consequently, interfere with the gene regulatory functions of CD44-ICD. The γ-secretase complex processes the precursor of amyloid protein (APP) and cleavages the intracellular domain of NOTCH signaling protein (NOTCH-ICD), analogously, to CD44-ICD. Several γ-secretase complex inhibitors (GSIs) have been developed and deployed for the treatment of Alzheimer’s with variable success (219) and there has been a repositioning of these molecules as candidate drugs against cancer and immune diseases. Since GSIs are also active inhibiting CD44-ICD cleavage (220), the CD44-ICD downstream signalling could be effectively affected and consequently, CD44-mediated cell migration and invasion mechanisms. Many of these inhibitors have been extensively tested in clinical trials and currently, it is an active development field. For example, the GSI PF-3084014 (Nirogacestat) was well tolerated in patients with advanced cancer and demonstrated promising clinical benefit in patients with refractory, progressive desmoid tumors who receive long-term treatment (NCT01981551) (194). Another of these GSIs, LY450139 (Semagacestat), was also well-tolerated and demonstrated evidence of clinical activity in heavily pretreated patients with breast cancer and leiomyosarcoma (NCT01695005) (195). In preclinical studies, the potent GSI BMS-906024 showed enhanced antitumor activity in combination with paclitaxel versus either drug alone, decreasing cell proliferation and increasing apoptosis in *in vivo* studies using cell line- and patient-derived lung adenocarcinoma xenografts (196). However, this approach has not been tested to date in sarcomas and may represent an attractive treatment strategy.

### 6.3 Blocking CD44 With Antibodies

Antibodies targeting CD44 are being tested in preclinical and clinical trials for many types of cancer. These antibodies can be conjugated with anti-tumor drugs to target cells overexpressing CD44 to favor the antitumoral drug uptake or act as blockers of CD44-mediated signalling. There have been developed several molecule-conjugated antibodies. The Roche radioimmunoconjugate RO5429083 (RG7356) antibody targets a conformational-dependent epitope of CD44 (in the constant region of the extracellular domain), and it is being tested in clinical trials to treat neoplasms (NCT01358903) and acute myelogenous leukemia (NCT01641250) (197, 198). Another anti-CD44 monoclonal antibody, called U36, labeled with indium-111, was tested in head and neck carcinoma xenografts that expressed CD44v6 isoform. The authors of this study suggested the possible use of this antibody as a cancer detection tool (199). Bivatuzumab mertansine is the combination of a humanized monoclonal antibody against CD44v6 and a cytotoxic agent (mertansine), which has been tested in a phase I trial in head and neck carcinoma (200, 201). However, the therapeutic response in these trials was variable and severe skin toxicity was detected due to the binding of the antibody to the CD44v6 isoform located on the keratinocyte’s membrane.

Alternatively, CD44 blocking antibodies have also been developed. H4C4, a monoclonal antibody that recognizes the extracellular domain of CD44 (from residue 69 to residue 90), reduced tumor growth, metastasis, and post-radiation tumor recurrence in human pancreatic mice xenografts (202). This antibody affected both bulk tumor cells and tumor initiating cells, decreasing the capacity of self-renewal and tumor initiation through STAT3 signalling inhibition and the downregulation of the stemness gene Nanog (202). The CD44 blocking monoclonal antibodies IM7 (that recognizes the constant region of the ECD, specifically residues 145-186) and KM201 (that binds to a region that is close to the HA binding domain) abrogate the VEGF secretion mechanism through the inhibition of the HA-CD44 signalling in human umbilical vein endothelial cells (203). Additionally, Uchino et al., demonstrated that the IM7 antibody decreased cell migration and invasion capacities in breast cancer cells (MCF-7-14 cells and its clone CL6, and MDA-MB-231 cells) using *in vitro* conditions (204). Therefore, there is an untested group of anti-CD44 blocking antibodies, which may have therapeutic potential in the context of sarcomas, but further research is needed.

### 6.4 Blocking CD44 With Peptides and Aptamers

Another therapeutic strategy could be based on the use of peptides and aptamers to block CD44 interactions or reduce CD44 expression. These molecules have a great affinity and specificity to target proteins and thus are excellent tools to inhibit their functions. Several peptides and RNA aptamers directed against CD44 have already been tested in many cancer models (221). The peptide PEP-1 (a short amphipathic peptide that blocks the interaction CD44/HA) reduced the CD44 expression levels in mice models of gastric cancer (205). PCK3145, a synthetic peptide corresponding to amino acids 31-45 of prostate secretory protein 94 (PSP94), was demonstrated to reduce bone metastases and prostate tumor growth tumor in rats injected with MAT-Ly-Lu-B-2 cell line (206). The mechanism of action of this antitumoral peptide is not completely understood, but it seems the peptide interferes with the tyrosine kinase activity associated with the VEGF signalling axis in endothelial cells inhibiting angiogenesis processes (*in vivo* testing with Mat Ly Lu rat prostate cancer cells) (207) and reduces the levels of plasma matrix metalloproteinase MMP-9 (a CD44 ligand). Annabi et al., studied the antitumoral effect of this peptide in the fibrosarcoma cell line HT1080 and showed that it inhibited cell adhesiveness to HA, laminin-1, and type-I collagen. PCK3145 inhibited the secretion of MMP-9 and triggered the shedding of the ectodomain of CD44 from the cell surface preventing the interaction of CD44 with MMP-9 (208). The RNA aptamer (Apt1) was used to functionalize the surface of PEGylated liposomes. This combination, Apt1-Liposome, allowed a better cellular uptake of the conjugated molecule in CD44 positive cell lines A549 (lung cancer) and MDA-MB-231 (breast cancer) (209). Finally, CD44-EpCAM (Epithelial Cell Adhesion Molecule) aptamer is a double-stranded RNA adaptor which acts blocking both, CD44 and EpCAM, simultaneously reducing tumor progression and promoting apoptosis, both *in vitro* and *in vivo* xenograft models of ovarian tumor cells (OVCAR8) (210).

### 6.5 CD44-Ligand Chemotherapy Delivery Systems

In addition, several strategies exploit the ability of CD44 to interact with a variety of extracellular proteins (25). For example, the interaction of CD44 with HA, its principal ligand, can be used to maximize the uptake of chemotherapy delivery systems on cells overexpressing CD44. Chemotherapeutic agent-encapsulated liposomes, or covalently bound HA-bioconjugates, were selectively taken up by tumor cells after systemic delivery and reduced specific gene expression in several xenograft models (222–224). Several of these innovative tools based on HA-functionalized liposomes have become an excellent CD44-mediated intracellular delivery system for doxorubicin (DOX) or photodynamic anticancer therapy in osteosarcomas: Chol-SS-mPEG/HA-L (211), HA-LsDOX (212), ALN–HA–SS–L–L/DOX (213), and HA-es-ZnPP (214). Chol-SS-mPEG/HA-L is an HA-coated redox responsive liposome, whose cytoplasmic drug release system is triggered by GSH. The HA-LsDOX liposomes mechanism consists in the release of the drug into the endoplasmic reticulum, promoting protein sulfhydration and ubiquitination, and activating CHOP-mediated pro-apoptotic signalling. ALN-HA-SS-L-L/DOX is a liposome with dual bone and CD44 targeting abilities and redox sensitivity whose efficacy appears to be increased by the co-administration of internalizing RGD (Arg-Gly-Asp motif). HA-es-ZnPP, a hyaluronan conjugated zinc protoporphyrin *via* an ester bond with a tumor environment-responsive mechanism, was tested *in vitro* and in a mouse sarcoma S180 solid tumor model. This nanoprobe for photodynamic anticancer approach combined with a xenon light source, demonstrated prolonged circulation time and enhanced cell permeability and retention. Consequently, an improved anticancer therapy effect without apparent side effects.

The micelles with HA functionalization have been used in other drug–gene combinations therapies to co-delivery the antitumor treatments (e.g., siRNA against overexpressed cancerous genes, anticancer drugs-Paclitaxel, doxorubicin, superparamagnetic iron oxide nanoparticles-SPIONs…) (225–228). The synergistic effect of these micelles on cell cytotoxicity was tested in the MDA-MB-231 breast cancer cell (229) and OVCAR8TR ovarian cancer cells (230, 231), both overexpressing CD44. Additionally, a dual-targeting platform for CD44 has already been tested in hepatic tumors (e.g., HA- glycyrrhetinic acid -conjugated polymer to target liver) (232, 233) and breast cancer (e.g., HA-folic acid micelles for cells overexpressing the folic acid receptor) (234). These multiple targeting strategies showed better consistency and increased cellular uptake, thus enhanced cytotoxicity. In the study performed by Xi Y et al. curcumin was used in a dual-targeting delivery therapy combining the active bone accumulating ability with tumor CD44 targeting capacity in alendronate-hyaluronic acid-octadecanoic acid (ALN-HA-C18) micelles (215). The curcumin loaded in ALN-HA-C18 micelles showed increased cytotoxic activity against MG-63 cells and reduced tumor growth in the osteosarcoma mouse model compared to the free drug. Nanoparticles labeled with CD133-EGFR aptamers were determined to inhibit tumor growth in the osteosarcoma mouse model (235). Thereby, novel combinations of dual-targeting such as CD133-CD44 aptamers, both overexpressed in osteosarcoma CSCs, are potentially susceptible to being tested in these malignancies. Overall, these innovative delivery packages showed an increased tissue specificity, enhanced anticancer effect and great potential in clinical developments and delivery systems, supposing a therapeutic alternative superficially explored in sarcomas.

In summary, these studies indicate that treatment with HA-functionalized liposomes or photodynamic therapy reduced tumor growth and increased survival time in xenograft models of osteosarcoma, becoming a promising osteosarcoma-targeted therapy. Beyond its biological properties, HA nanomedicine is limited by the reduced methods for chemical conjugation, the fluctuant cellular uptake caused by the chemical characteristics of HA, and the unavoidable biological degradation when it enters *in vivo* (228). Therefore, the effects of these limitations need to be further studied to ensure minimum adverse responses and maximum clinical efficacy.

### 6.6 Blocking CD44 With CD44 Ligands

Finally, another way to block the CD44 function is by blocking CD44-HA interactions. The soluble CD44 ectodomain has been demonstrated to compete with the CD44-HA interaction on malignant cells, inhibiting tumor progression and invasiveness (236). Conversely, another strategy is to use HA-oligomers to block CD44-HA interactions. HA oligomers are small molecule chains composed of a variable number of units (6-12 mers). These molecules have been shown to suppress signalling pathways involved in drug resistance against different therapeutic agents (237, 238). Several studies have shown that HA disaccharides bind CD44, competing by and displacing the biological HA polymer (239, 240) and can inhibit HA synthesis (241). Despite its therapeutic potential there are few studies addressing the therapeutic possibilities of these molecules in sarcomas. Hosono et al., studied the effect of hyaluronan oligosaccharides on tumor growth in murine (LM-8) and human (MG-63) osteosarcoma cells. Hyaluronan octamers promoted apoptosis and reduced both cell viability, cell proliferation and cell motility, and decreased the retention of endogenous HA. The intratumoral injection of these molecules in xenograft models produced a reduction of HA accumulation in the tumor, suppressing the dissemination of tumor cells to the lung (216). A similar effect has been observed in other cancers such as melanoma, carcinoma, or glioma (94, 217). HA oligomers therapies provide a viable alternative for blocking the CD44-HA interactions in sarcomas which has been only superficially explored, so that clinical trials should be performed to confirm this antitumoral approach.

## 7 Conclusions

CD44 has a key role, along with ECM components, in sarcoma tumor development, metastasis, and drug resistance. Understanding CD44 signalling is crucial to comprise the multifactorial role of this protein in several processes, such as activation of different pathways, upregulation of several genes, or modification of cytoskeletal structures that promote EMT mechanisms, among others.

MSCs are thought to give rise to sarcomas. As consequence, several molecular mechanisms, transcription factors, metabolic pathways, and cell markers are shared among MSCs, CSCs and sarcomas. CD44, in this context, provides the elements to maintain the stemness and to take advantage of the tumoral microenvironment in hypoxia conditions. Studies focused on MSCs, and sarcomas have shown CD44 to be highly expressed in these tissues compared to mature cells. Thus, it has been proposed that during cell differentiation, expression of CD44 could be downregulated, and conversely, high levels of CD44 are necessary to maintain the cells in a stemness state.

Further research involving the sarcoma functional mechanisms could be really enriching in the characterization context of sarcoma, as well as, in the therapeutic approaches targeting CD44. Some of the innovative treatment tools such as peptides, aptamers, HA oligomers, pharmacological substances, and chemotherapy delivery systems, among others, have been developed from the discovery and well-understanding of the CD44 structure, the signalling pathways and the interactions involved.

The data from the characterization of osteosarcomas have revealed that CD44 (concretely CD44v6 isoform) is a potential and promising marker for prognosis and diagnosis. There is a low number of detailed studies deciphering the prognostic/diagnostic value of CD44 in sarcomas (excluding osteosarcoma which is one of the most studied and common sarcomas). In addition, sarcoma-specific studies involving a greater number of patients are necessary to confirm the influence of CD44 variant expression on the patients’ prognosis (regardless of the utility of CD44v6 as a prognostic marker in osteosarcoma). The complexity and distinct molecular origin of the different sarcoma subgroups have made impossible to apply a unified characterization or prognosis criterion, and therefore they should be studied separately. In addition, the reduced number of studies focused on CD44, and the low number of patients involved in the different sarcoma subgroups have produced inconclusive results. As consequence, the combination of different characterization markers will be more precise and will provide relevant information to determine patients’ outcomes, neither than the use of CD44 individually.

To conclude, more research about the contribution of CD44 in sarcomas could contribute to understand the pathogenesis of these rare cancers, and evolve towards novel therapies and future clinical trials.

## Author Contributions

Writing original draft preparation, EF-T; writing - review and editing, EF-T, RM-FM, and JA; supervision, JA; funding acquisition, JA. All authors have read and agreed to the published version of the manuscript.

## Funding

This review was funded by the Instituto de Salud Carlos III, grant numbers PI20CIII/00020, DTS18CIII/00005, Asociacion Pablo Ugarte, grant numbers TRPV205/18; Asociación Candela Riera, Asociación Todos Somos Iván & Fundación Sonrisa de Alex, grant numbers TVP333-19, TVP-1324/15; ASION, grant number TVP141/17. R.M.M-FdM is supported for a grant of the Spanish Center for Biomedical Network Research on Rare Diseases (CIBERER).

## Conflict of Interest

The authors declare that the research was conducted in the absence of any commercial or financial relationships that could be construed as a potential conflict of interest.

## Publisher’s Note

All claims expressed in this article are solely those of the authors and do not necessarily represent those of their affiliated organizations, or those of the publisher, the editors and the reviewers. Any product that may be evaluated in this article, or claim that may be made by its manufacturer, is not guaranteed or endorsed by the publisher.
